# Non-anticoagulant heparins: glycan-mediated immune modulation and therapeutic applications

**DOI:** 10.3389/fimmu.2026.1855688

**Published:** 2026-07-21

**Authors:** Yaner Gu, Qiong Wang, Juan Fang, Jing Chen, Kaiting Hong, Zhanwei Zhang, Syed Shams Ul Hassan, Yin Chen, Xuqian Zhang

**Affiliations:** 1 Zhoushan Maternal and Child Care Hospital, Zhoushan, China; 2 School of Food and Medicine, Zhejiang Ocean University, Zhoushan, China

**Keywords:** clinical applications, drug delivery systems, mechanism, non-anticoagulant heparins, structure-activity relationship

## Abstract

Non-anticoagulant heparins (NAHs) are modified derivatives of heparin that retain many of the biological activities of the parent compound while exhibiting reduced or absent anticoagulant effects. These modifications have unlocked new therapeutic potentials in various diseases, including inflammatory disorders, cancer, sepsis, and viral infections. This review explores the chemical modifications of NAHs, their mechanisms of action, structure-activity relationships, and therapeutic applications. NAHs exert anti-inflammatory, anticancer, antiviral, and vascular protective effects by interacting with key proteins like heparanase, P-selectin, and HMGB1. Furthermore, their application in drug delivery systems, such as hydrogels and nanoparticles, enhances localized therapeutic efficacy. The promising results from preclinical and clinical studies position NAHs as a valuable class of drugs with diverse therapeutic applications, particularly in oncology, infectious diseases, and critical care.

## 1 Introduction

**Figure 1:**
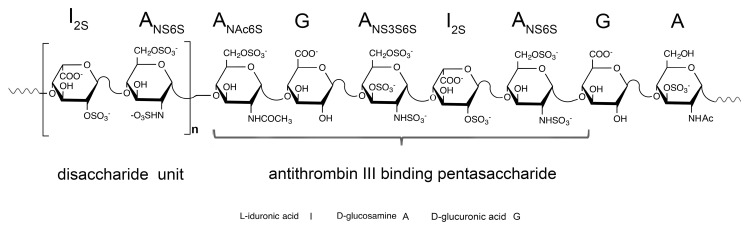
Structure of heparin. The repeating disaccharide unit consists of uronic acid (either L-iduronic acid, IdoA, or D-glucuronic acid, GlcA) and D-glucosamine (GlcN), linked by α (1→4) glycosidic bonds. Sulfation occurs at multiple positions: N-sulfation (N-S) on glucosamine, and O-sulfation at the 2-O-position of uronic acid and the 6-O-position of glucosamine. The unique pentasaccharide sequence (shown in the dashed box) constitutes the antithrombin III (AT-III) binding site, which is critical for the anticoagulant activity of unfractionated heparin. NAHs are generated by chemical modifications that disrupt this pentasaccharide motif while preserving other biological activities. This image is created by the authors using KingDraw.

The history of heparin dates back more than a century, when Jay McLean and William Henry Howell first isolated this glycosaminoglycan (GAG) from canine liver in 1916, with subsequent collaborative work leading to its purification and naming as “heparin” (1). Its powerful anticoagulant activity quickly established the compound as an essential therapeutic agent for thromboprophylaxis and embolism prevention. Structurally, heparin is a linear, highly sulfated polysaccharide composed of repeating disaccharide units of uronic acid (either iduronic acid or glucuronic acid) and glucosamine. These units are variably sulfated at multiple positions (*N*-, 2-*O*-, and 6-*O*-sulfation), giving the molecule dense negative charges and strong affinity for various proteins (**Figure 1**) (2).

The anticoagulant properties of heparin depend largely on its ability to bind antithrombin III (AT-III), a key regulator of the coagulation cascade. Within the heparin polymer, a unique pentasaccharide motif engages AT-III and triggers a conformational rearrangement that markedly potentiates the inhibitor’s activity against thrombin (factor IIa) and factor Xa (**Figure 1**), two critical enzymes in the coagulation cascade (3). This interaction accelerated the neutralization of these coagulation factors by up to 1,000-fold, effectively preventing fibrin clot formation (4).

Despite its widespread use, particularly in high-risk surgical and medical settings, the therapeutic applications of heparin extended far beyond its anticoagulant properties. Recent studies have uncovered its non-anticoagulant effects, such as anti-inflammatory, anticancer, antiviral, and vascular protective properties, which have significantly expanded its clinical utility (5–12).

This review systematically examines the preparation, biological activities, and mechanisms of action of NAHs, with particular emphasis on quantitative structure-activity relationships that link specific chemical modifications to selective target recognition. Previous reviews have explored the therapeutic potential of NAHs from distinct angles: Cao et al. (13) focused on their application in COVID-19; Lanzi et al. (14) discussed their role in sarcoma; Sultana et al. (15) and Wang et al. (16) provided overviews of antiviral and anticoagulant applications; and Zeng et al. (17) summarized general anti-inflammatory and anticancer mechanisms. While these contributions have advanced the field, they largely treat these therapeutic areas—anti-inflammatory, anticancer, vascular protective, and antiviral effects—as separate topics, each considered in isolation. In contrast, this review provides an integrated framework that bridges chemical structure-activity relationships (SAR) with molecular mechanisms of immune modulation, presenting these diverse therapeutic activities as a cohesive network of glycan-mediated protein interactions involving shared targets such as heparanase, P-selectin, and HMGB1. Furthermore, we critically assess the translational barriers highlighted by recent clinical failures (RASTEN, FRAGMATIC, necuparanib, PI-88) and discuss how target-selective NAH design and delivery innovations may overcome these obstacles. By unifying chemical, mechanistic, and clinical perspectives, this review aims to provide a practical roadmap for developing NAH-based therapies with improved target selectivity and reduced bleeding risk.

## 2 Preparation of non-anticoagulant heparins

### 2.1 Chemical modification techniques

**Figure 2:**
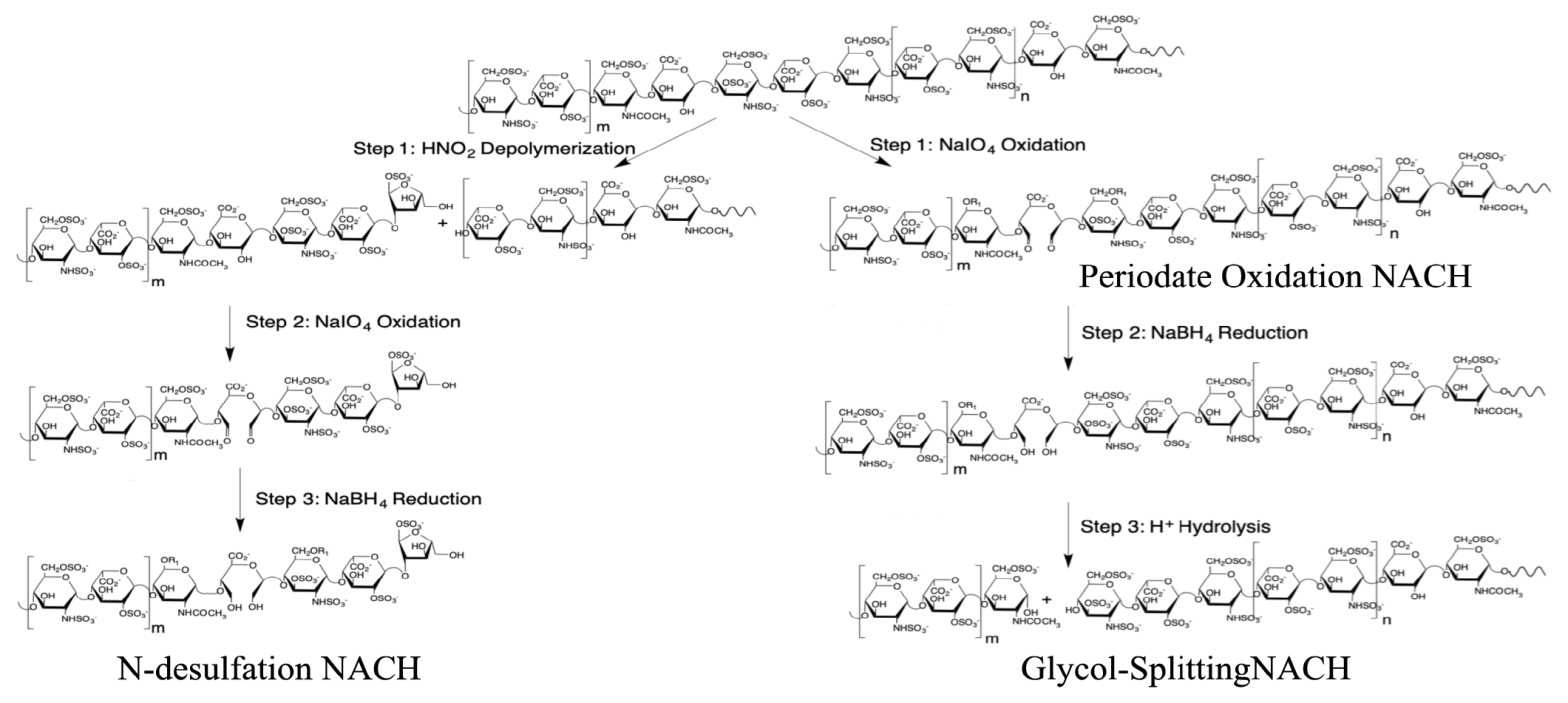
Preparation of non-anticoagulant heparins. Three chemical strategies are shown: (1) Periodate oxidation cleaves the C2-C3 bond of unsulfated uronic acids, generating flexible glycol-split (gs) residues and disrupting the AT-III binding site. (2) N-desulfation removes N-sulfate groups from glucosamine, which may be followed by N-acetylation. (3) Glycol-splitting (periodate oxidation followed by reduction) converts non-sulfated uronic acids to gs residues. This image is created by the authors using KingDraw.

The preparation of NAHs involved specific chemical modifications designed to remove the anticoagulant activity of heparin while retaining its other therapeutic benefits. Key strategies include periodate oxidation, N-desulfation, and glycol-splitting (**Figure 2**) (18, 19).

#### 2.1.1 Periodate oxidation

Among the various chemical modification strategies, periodate oxidation remains a widely employed approach for generating NAHs. This technique selectively targets vicinal diol groups located on unsulfated glucuronic acid (GlcA) residues within the pentasaccharide domain responsible for AT-III binding. Cleavage of these diol moieties effectively abolishes the conformational integrity required for AT-III recognition, thereby eliminating anticoagulant activity (20). This modification eliminated the anticoagulant activity while preserving the molecule’s ability to interact with other important biological targets, such as heparanase and P-selectin, which are involved in inflammation and cancer progression (21).

#### 2.1.2 N-desulfation

Another method, N-desulfation, led to the removal of sulfate groups from the glucosamine residues in heparin (22). For example, the use of enzymes such as N-sulfatases that specifically targeted the sulfate groups attached to the nitrogen of the glucosamine residues (23, 24). These enzymes cleaved the sulfate groups without affecting the backbone of the heparin molecule. Since sulfate groups were essential for activating antithrombin III, their removal significantly reduced heparin’s anticoagulant effects, allowing it to retain its biological activities related to immune modulation, cancer metastasis, and inflammation (25).

#### 2.1.3 Glycol-splitting

Glycol-splitting was a chemical modification that treating heparin with sodium periodate to selectively cleave the C2-C3 vicinal diol moiety in the unsulfated glucuronic acid (GlcA) and iduronic acid (IdoA) residues of the polysaccharide. This treatment resulted in the formation of a ring-opened dialdehyde residue, which was subsequently reduced with sodium borohydride. Following reduction, the product was cleaved with acid to yield NAHs with a reduced molecular weight (26).

Nitrous acid depolymerization of the porcine intestinal heparin was another method to prepare NAHs (27). Nitrous acid specifically cleaved the glycosidic bonds between glucosamine (GlcN) and uronic acid. The reaction involved the formation of an N-nitroso derivative at the amine group of the glucosamine (28). The resulting fragments retained an intact AT-III binding site and possess clinically significant anti-Xa activity (typically 70–120 IU/mg). Therefore, nitrous acid depolymerization as low molecular weight heparins (LMWHs) alone does not produce true NAHs. To convert LMWHs into NAHs, additional chemical modifications—such as periodate oxidation and glycol-splitting—are required to disrupt the AT-III binding site. These smaller fragments had disrupted AT-III binding site and exhibited reduced anticoagulant activity but retained other therapeutic properties, such as anti-inflammatory and anticancer effects. The reduced molecular size improved their bioavailability and tissue penetration, making them suitable for localized treatment of diseases like cancer and inflammatory conditions (29).

### 2.2 Structural characterization of non-anticoagulant heparins

Although both heparin and its derivatives could bind to various heparin-binding proteins, such as P-selectin and heparanase, the key distinction lied in their anticoagulant activity. Unfractionated Heparin (UFH) is a highly charged, large molecule that strongly activated AT-III, leading to its anticoagulant effects. In contrast, NAHs are engineered to avoid this interaction, making them particularly useful in therapeutic areas focused on inflammation, cancer progression, and immune modulation, while minimizing the risk of bleeding (30, 31).

Advanced characterization techniques, including high-performance liquid chromatography (HPLC) and nuclear magnetic resonance (NMR) spectroscopy, were used to confirm structural changes and to verify that the desired modifications are achieved without compromising therapeutic properties. NMR spectra could provide information on the persistence of key functional groups, such as the sulfation pattern of the heparin backbone can be confirmed by analyzing the distinct chemical shifts corresponding to different sulfate positions on the sugar residues (32).

Additionally, HPLC-MS assay had been developed to analyze the 3-*O*-sulfo group-containing tetrasaccharides, allowing for precise measurement of the AT-III-binding site variants (33). In detail, heparin was often treated with heparin lyase II, an enzyme that cleaves the heparin polysaccharide chains into tetrasaccharide fragments (34). These fragments were resistant to further enzymatic digestion and contain the essential 3-*O*-sulfo groups involved in the antithrombin-binding interaction. This approach provided insights into the natural variability of the antithrombin-binding region across different heparin sources and was crucial for distinguishing NAHs from normal heparin (35).

This source-dependent structural variability extends beyond the AT-III-binding site to the entire heparin chain. Pharmaceutical heparin is primarily derived from porcine intestinal mucosa, but bovine lung and bovine intestinal heparins are also commercially available. These sources differ in molecular weight distribution, sulfation pattern, and anticoagulant activity (36, 37). Porcine heparin contains a higher proportion of the antithrombin-binding pentasaccharide and exhibits higher specific activity than bovine heparins. For NAH preparation, source heterogeneity introduces additional batch-to-batch variability that must be considered during manufacturing and quality control.

## 3 Biological activities of non-anticoagulant heparins

### 3.1 Anti-inflammatory properties

One of the most significant therapeutic properties of NAHs was their ability to modulate inflammation. Cell-based and animal studies have demonstrated that these derivatives acted by inhibiting leukocyte adhesion to endothelial cells, a key event in the inflammatory response. By binding to P-selectin and inhibiting heparanase activity, NAHs prevented the interaction between leukocytes and endothelial cells, reducing immune cell infiltration at sites of inflammation (38, 39).

**Figure 3:**
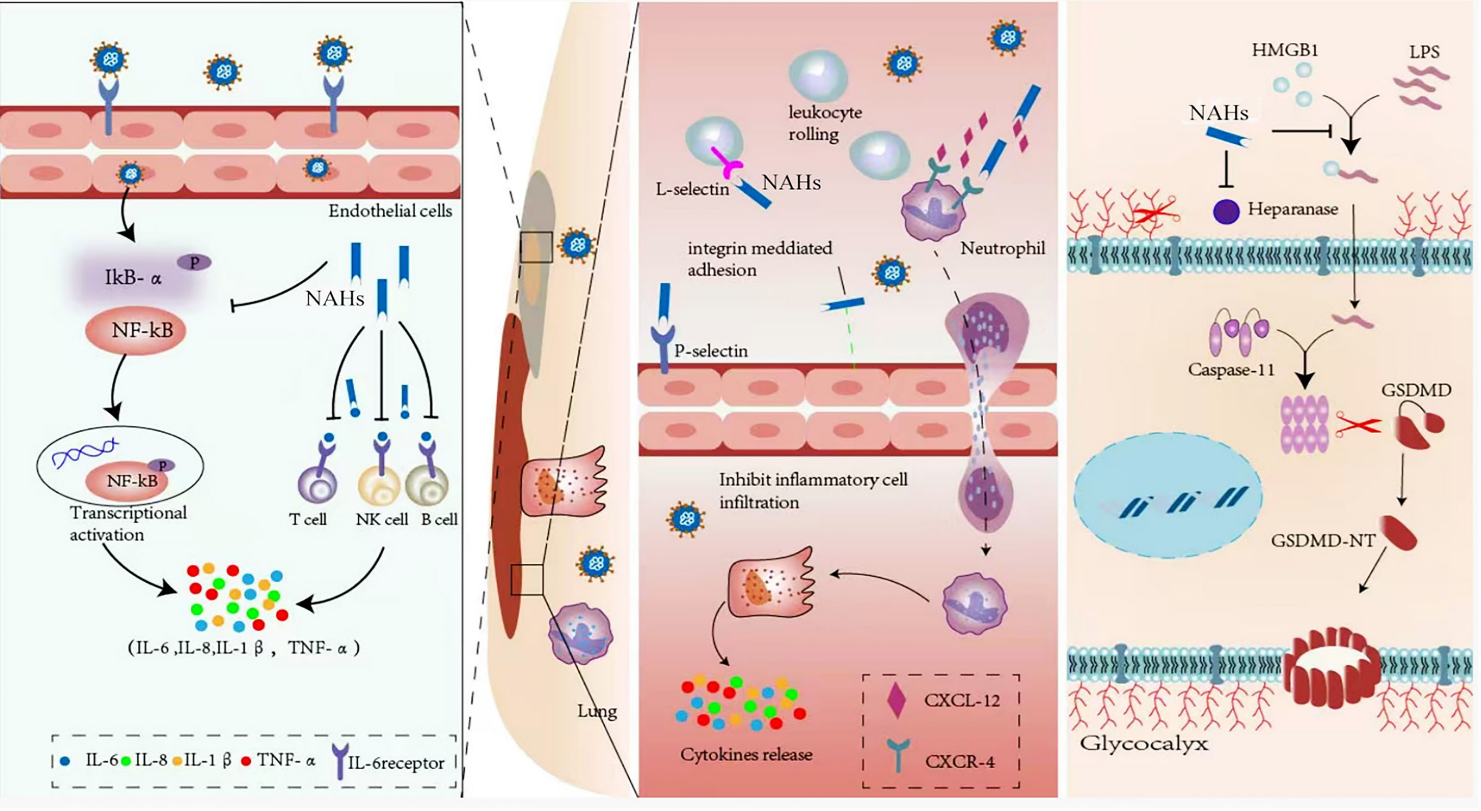
Schematic illustration of anti-inflammatory activity of NAHs. The anti-inflammatory effects include three general mechanisms: NAHs inhibit (1) pro-inflammatory cell infiltration by suppressing neutrophil and leukocyte recruitment and adhesion; (2) IL-6 function through NF-κB inhibition and direct IL-6 binding; and (3) HMGB1 activation. The schematic was designed based on the mechanistic framework described in the literature (40). This image is created by the authors using SciDraw.

Additionally, NAHs suppressed the release of pro-inflammatory cytokines including TNF-α, IL-1β, and IL-6, and inhibit the activation of HMGB1 (**Figure 3**), thereby mitigating inflammatory responses in conditions such as sepsis and autoimmune diseases (41, 42).

Beyond these well-characterized pathways, emerging evidence suggests that NAHs may also modulate broader inflammatory networks. Heparin and its derivatives have been shown to interfere with complement activation, particularly through binding to complement components such as C1q and C3, thereby attenuating complement-mediated tissue injury (43). Additionally, NAHs can bind to a range of chemokines including CXCL8 (IL-8) and CCL2 (MCP-1), disrupting their chemotactic gradients and reducing the recruitment of neutrophils and monocytes to inflammatory sites (44). This interference with chemokine–glycosaminoglycan interactions represent an additional layer of anti-inflammatory activity that complements the direct inhibition of cytokine release and leukocyte adhesion. Furthermore, heparin has been shown to modulate the NF-κB signaling pathway in endothelial cells and monocytes, reducing the transcription of multiple pro-inflammatory genes (45). These pleiotropic effects on the complement, chemokine, and transcription factor networks further underscore the therapeutic potential of NAHs in inflammatory diseases, particularly in conditions where multiple inflammatory mediators converge, such as sepsis, acute respiratory distress syndrome, and chronic autoimmune disorders.

### 3.2 Anticancer effects

NAHs exhibited potent anticancer effects by targeting multiple pathways involved in tumor progression. They inhibited tumor metastasis by preventing heparanase, an enzyme that cleaved heparan sulfate chains within the extracellular matrix (ECM), which was essential for cancer cells to invade and spread. By inhibiting heparanase activity, NAHs reduced tumor cell migration and invasion (46, 47).

In addition, NAHs had been shown to suppress angiogenesis, the process by which tumors form new blood vessels to supply nutrients and oxygen. By blocking key signaling molecules like vascular endothelial growth factor (VEGF) and Basic Fibroblast Growth Factor (bFGF), NAHs prevented the formation of new blood vessels, effectively starving the tumor and limiting its growth (17, 48).

Beyond its direct effects on tumor cells and angiogenesis, the tumor microenvironment contains various immune cell populations that influence cancer progression. Among these, tumor-associated macrophages (TAMs) are particularly abundant and typically exhibit an M2-like immunosuppressive phenotype that promotes tumor growth and immune evasion. Recent evidence suggests that NAHs can modulate this polarization state. Using a murine macrophage cell line, Zhu et al. (49) demonstrated that N-desulfated and reacetylated heparin derivatives promote a shift from M2-like to M1-like polarization. This effect was mediated by the STAT6 pathway and was associated with reduced M2 markers (Arg-1, CD206) and increased pro-inflammatory cytokines (IL-12, TNF-α). These findings suggest that certain NAH modifications may help reprogram the immunosuppressive tumor microenvironment, although direct evidence in tumor-bearing hosts remains to be established.

**Figure 4:**
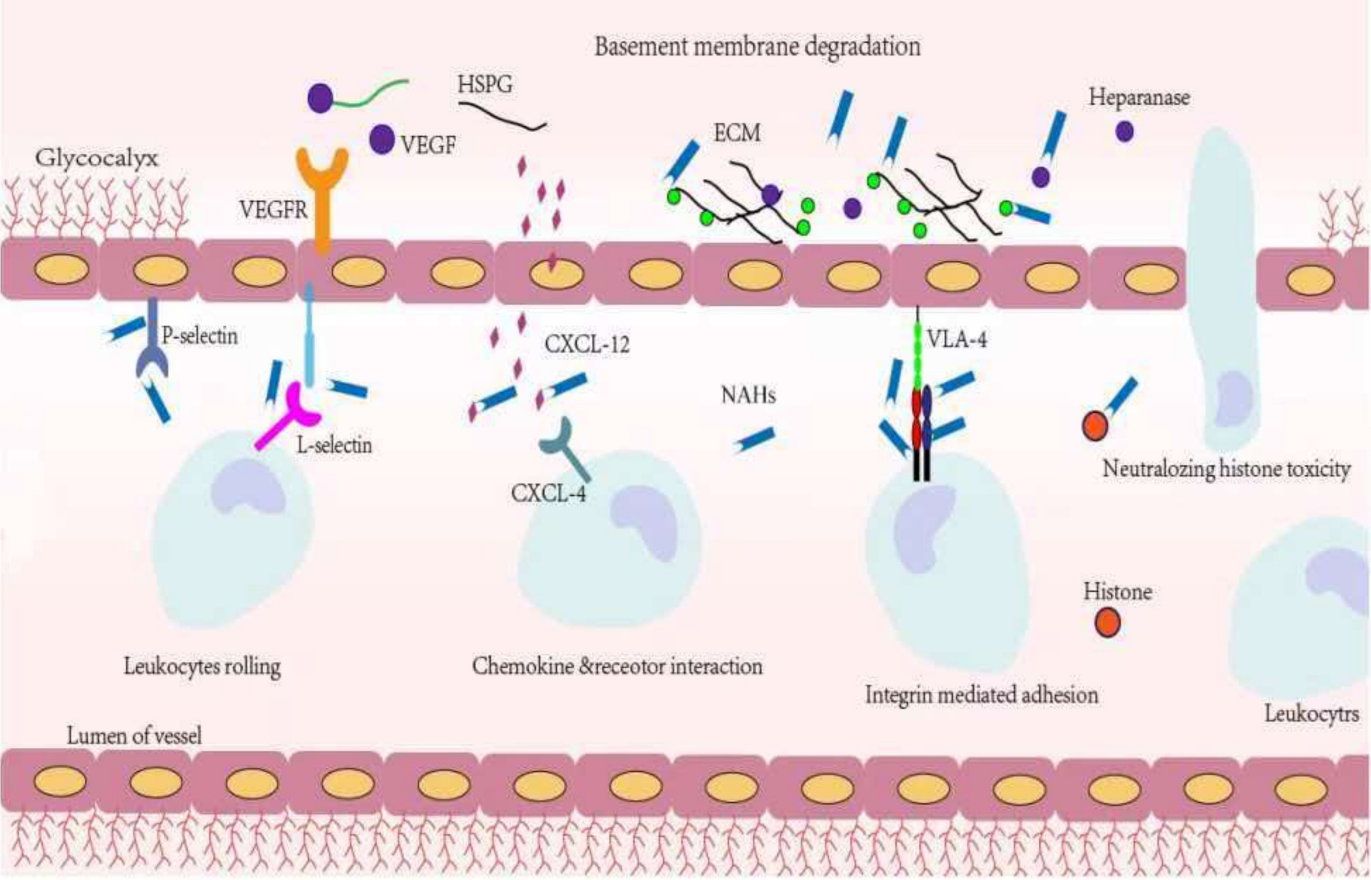
Schematic illustration of anti-tumor activity of NAHs. NAHs inhibit (1) heparanase-mediated ECM degradation, (2) VEGF/FGF-driven angiogenesis, and (3) tumor-associated inflammation. This image is created by the authors using SciDraw.

In addition, heparin and its derivatives can neutralize damage-associated molecular patterns (DAMPs) such as extracellular histones, which are released from necrotic and apoptotic cells within the tumor microenvironment and promote inflammation, endothelial injury, and thrombosis (50). Heparin binds to positively charged histones through electrostatic interactions, an effect that is independent of its anticoagulant activity and is retained by non-anticoagulant derivatives (38). By mitigating DAMP-mediated inflammation, NAHs may indirectly suppress tumor progression, though this mechanism is primarily anti-inflammatory rather than directly immunomodulatory.Early studies demonstrated that the glycol-split heparin derivative had shown promise in cancer models, significantly reducing tumor growth and metastasis in preclinical studies. These preclinical findings suggest that NAHs may have potential value as part of combination therapies for cancer treatment. However, several caveats should be noted. First, most of these studies were conducted in murine xenograft models, which may not fully recapitulate human tumor biology or the tumor microenvironment. Second, the effective doses used in animal studies (typically 10–20 mg/kg daily) are often substantially higher than those achievable in patients without bleeding risk, particularly for anticoagulant LMWHs. Third, as discussed, clinical translation of these promising preclinical results has been challenging, with several Phase III trials failing to demonstrate survival benefit. Therefore, while NAHs hold promise, rigorous clinical evaluation is required to establish their therapeutic efficacy in cancer patients (**Figure 4**) (51).

### 3.3 Antiviral activity

Preclinical evidence suggests that heparin derivatives, including certain NAHs, may exert antiviral effects. Studies using unfractionated heparin (UFH) and LMWHs have demonstrated that these compounds can block viral entry by binding to viral surface glycoproteins (e.g., the spike protein of SARS-CoV-2), thereby preventing attachment to host cell heparan sulfate (HS) or ACE2 receptors. Surface plasmon resonance (SPR) studies have demonstrated that UFH binds to the SARS-CoV-2 spike glycoprotein with high affinity (Kd ~40–70 pM for the receptor-binding domain), while LMWHs show weaker binding (Kd ~10–100 nM) (52, 53). Although direct binding data for NAHs are limited, their structural similarity to UFH suggests that they may retain significant binding affinity, a hypothesis that requires experimental validation. This inhibitory effect is independent of AT-III binding and is fully retained in NAHs (16).

Beyond entry inhibition, heparin derivatives suppress heparanase activity, an enzyme that promotes viral egress and cell-to-cell transmission. They also neutralize pro-inflammatory cytokines and damage-associated molecular patterns (DAMPs) such as HMGB1, thereby mitigating the hyperinflammatory response (“cytokine storm”) associated with severe viral infections like COVID-19. While these effects have been well documented for UFH and LMWH, preliminary studies suggest that NAHs retain these activities due to their structural similarity to heparin. (40, 54).

Importantly, while NAHs are engineered to have markedly reduced antithrombin III (AT-III) binding and minimal anticoagulant activity (typically anti-Xa <10 IU/mg), they are not completely devoid of anticoagulant effects. This residual, weak anticoagulant activity—though clinically insignificant in terms of bleeding risk—may still contribute to the prevention or attenuation of virus-associated thrombotic complications, such as the pulmonary microthrombi and disseminated intravascular coagulation observed in severe COVID-19. In this context, the weak anti-Xa/IIa activity retained by certain NAH derivatives could provide a dual benefit: suppressing viral entry and inflammation while mildly modulating coagulation without increasing bleeding risk. This stands in contrast to conventional LMWHs (e.g., enoxaparin) and UFH, which possess potent AT-III-mediated anticoagulant activity (anti-Xa 70–200 IU/mg) that, while therapeutically useful, carries a significant bleeding risk.

**Figure 5:**
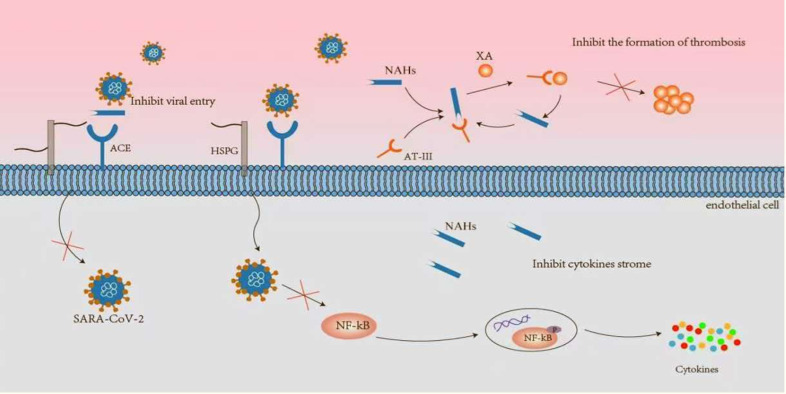
Summary of the potential antiviral mechanisms of NAHs: 1. Reducing viral entry, NAHs interacted with the SARS-CoV-2 spike glycoprotein to inhibit viral entry. 2. Inhibition of heparanase activity, NAHs suppress heparanase activity, which is elevated in COVID-19 and correlates with disease severity. 3. Neutralization of chemokines and cytokines, NAHs bound to chemokines and cytokines, including those involved in the cytokine storm associated with COVID-19. 4. Unlike conventional UFH or LMWHs, NAHs are engineered to have markedly reduced antithrombin III (AT-III) binding and minimal anticoagulant activity. However, certain NAH derivatives may retain weak anticoagulant activity through alternative mechanisms or at very high concentrations, which, though clinically insignificant in terms of bleeding risk, could still modulate coagulation in specific pathological contexts. The schematic was designed based on the mechanistic framework described in the literature (54). This image is created by the authors using SciDraw.

NAHs acted as broad-spectrum antivirals by blocking viral entry and enhancing immune defense, offering therapeutic promise against both established and emerging viral threats (**Figure 5**) (15). In addition to directly blocking viral entry, NAHs can mitigate the severe inflammatory consequences of viral infection by neutralizing pro-inflammatory chemokines and cytokines. Severe viral infections, including COVID-19, are often characterized by a “cytokine storm”—an excessive release of inflammatory mediators such as interleukin-6 (IL-6), tumor necrosis factor-α (TNF-α), and chemokines like CXCL8 (IL-8) and CXCL10 (IP-10) (55). These cytokines promote leukocyte recruitment, vascular leakage, and tissue damage, contributing to acute respiratory distress syndrome (ARDS) and multi-organ failure.

Heparin and its derivatives bind to a wide range of cytokines and chemokines through electrostatic interactions between their negatively charged sulfate groups and positively charged amino acid residues on these proteins (43). This binding can neutralize cytokine activity by preventing interaction with their cognate receptors. For example, heparin has been shown to bind IL-6 with high affinity (Kd in the nanomolar range) and to inhibit IL-6-mediated signaling (56). Similarly, heparin binds CXCL8 (IL-8) and disrupts its chemotactic gradient, thereby reducing neutrophil recruitment to sites of inflammation (57, 58). Although most of these studies were performed with UFH or LMWH, NAHs retain this cytokine-binding capacity due to their preserved sulfation patterns (5). By neutralizing pro-inflammatory cytokines, NAHs may attenuate the systemic inflammatory response during severe viral infections, complementing their direct antiviral effects.

### 3.4 Vascular protection

NAHs presented significant vascular protection by enhancing endothelial nitric oxide synthase (eNOS) activity. Nitric oxide (NO) is a potent vasodilator that helps regulate vascular tone and blood pressure. By increasing NO production, NAHs improve vascular function and reduce the risk of vascular dysfunction seen in conditions like atherosclerosis and hypertension (59).

In conditions such as ischemia-reperfusion injury (IRI) and septic shock, where endothelial dysfunction played a key role, NAHs help restore normal vascular permeability and blood flow, preventing further tissue damage and promoting recovery (60).

In summary, by promoting NO-mediated vasodilation and stabilizing endothelial function, NAHs protect against vascular injury and dysfunction, underscoring their potential in cardiovascular and critical care contexts.

## 4 Mechanisms of action of non-anticoagulant heparins

### 4.1 Molecular interactions

The therapeutic effects of NAHs are primarily due to their ability to interact with a variety of heparin-binding proteins involved in inflammation, cancer, and immune regulation. NAHs can modulate the function of key proteins such as heparanase, P-selectin, chemokines, HMGB1, and VEGF, which are crucial for cell adhesion, immune response, tumor progression, and angiogenesis (61).

#### 4.1.1 Heparanase inhibition

One of the most important interactions is with heparanase (62). Heparanase is an enzyme that degrades heparan sulfate (HS) chains, a key component of heparan sulfate proteoglycans (HSPGs) on the cell surface and in the extracellular matrix (ECM). HS plays a critical role in regulating tumor cell metastasis. In cancer, heparanase is upregulated and contributes to tumor progression by breaking down HS, which facilitates the release of growth factors, such as fibroblast growth factor-2 (FGF-2) and VEGF, from the ECM (63). These growth factors, once freed, can bind to their respective receptors and activate mitogenic signaling, promoting tumor growth and angiogenesis. Non-anticoagulant heparin can inhibit heparanase activity, preventing the degradation of HS. For example, the glycol-split N-acetylated heparin derivative roneparstat exhibits an IC_50_ of approximately 3 nM against human heparanase, which is comparable to or better than that of UFH (IC_50_ ~10–50 nM) (64, 65). Similarly, the 2-O,3-O-desulfated heparin derivative ODSH has been reported to inhibit heparanase with an IC_50_ in the low micromolar range (66).

This inhibition disrupts the availability of growth factors, effectively halting their activation and signaling, which is critical in tumor growth and metastasis. By inhibiting heparanase, NAHs prevent the breakdown of the ECM, thus inhibiting cancer cell invasion and metastasis (67).

In addition, heparanase and the endothelial glycocalyx have been linked to certain inflammatory responses. Enhanced heparanase activity contributes to the formation of proinflammatory glycocalyx. Heparanase-generated heparan sulfate fragments promote inflammation in the extracellular milieu. Under such conditions, the glycocalyx undergoes structural alterations that facilitate the binding of chemokines, selectins, and integrins expressed by leukocytes (68). Glycosaminoglycans on endothelial cells regulate bradykinin signaling; degraded heparin accelerates kininogen cleavage to bradykinin. Thus, non-anticoagulant heparin may reduce slow-kinin production—via heparanase inhibition and sequestration of high-molecular-weight kininogen—and in turn lessen local inflammation (69). Heparin and LMWH might also exert effects by blocking complement activation.

#### 4.1.2 Selectin and integrin antagonism by heparins

P-selectin is a cell-surface adhesion protein expressed on activated endothelial cells and platelets. It mediated leukocyte and platelet tethering to the endothelium, thereby driving inflammatory and thrombotic responses. NAHs bound and antagonized P-selectin, disrupting leukocyte–endothelium interactions and consequently limiting immune-cell infiltration at inflamed sites (70). The binding affinity of heparin derivatives to P-selectin varies with molecular weight and sulfation pattern. UFH binds to P-selectin with a Kd of approximately 0.1–1 μM, while LMWHs exhibit weaker affinity (Kd ~5–20 μM) due to reduced chain length. Among NAHs, glycol-split derivatives retain significant P-selectin inhibitory activity, with IC_50_ values ranging from 20–60 μg/mL in cell adhesion assays (71). In cancer, P-selectin promoted the formation of tumor cell–platelet aggregates (emboli), which shield circulating tumor cells from immune surveillance and facilitate hematogenous dissemination. By blocking P-selectin–dependent adhesion, heparins—including NAHs—impaired tumor cell binding to platelets and leukocytes, reducing survival of circulating tumor cells and lowering metastatic potential (72). Multiple studies reported that NAHs could suppress metastasis at least as effectively as standard heparin, indicating that anticoagulant activity was not required for the antimetastatic effect (71).

Although P- and L-selectin have been the classical focus of heparin-mediated interference with leukocyte adhesion, emerging evidence from the Bendas group establishes that integrins represent an independent, non-anticoagulant target family of heparins. Specifically, the integrin VLA-4 (α4β1) was identified as a direct binding partner of unfractionated heparin (UFH) and tinzaparin, with low-micromolar dissociation constants, whereas the synthetic pentasaccharide fondaparinux — despite full anticoagulant activity via antithrombin—is completely inactive in this context (73, 74). This dissociation demonstrates that integrin antagonism is separable from anticoagulation. Moreover, the matricellular protein Cyr61 (CCN1) was identified as an additional heparin target that modulates VLA-4-dependent melanoma cell adhesion and migration (75). Beyond VLA-4, the integrin αIIbβ3 has also been implicated as a direct target of heparins, further expanding the integrin axis. Collectively, these findings indicate that heparins and their non-anticoagulant derivatives can modulate immune cell trafficking and tumor cell adhesion through integrin-directed mechanisms, complementing the well-established selectin pathway (76, 77).

The functional relevance of VLA-4 antagonism extends beyond direct adhesion blockade. VLA-4 plays a central role in leukocyte recruitment to inflammatory sites and micrometastatic niches, as well as in tumor immune evasion and chemoresistance (78). By targeting VLA-4, heparins and their non-anticoagulant derivatives may therefore modulate not only tumor cell adhesion but also the recruitment of tumor-associated leukocytes that shape the metastatic microenvironment. This immune-modulatory angle aligns with the broader theme of the manuscript, positioning integrin-directed NAHs as potential tools for interfering with both cancer cell dissemination and host–tumor interactions.

#### 4.1.3 HMGB1 modulation

NAHs also interacted with high mobility group box 1 protein (HMGB1), a protein released during cellular stress or damage and acts as a danger signal to activate the immune system. HMGB1 is a highly conserved non-histone nuclear protein. Within the nucleus, HMGB1 binds to various sequence-independent DNA regions, thereby regulating nuclear homeostasis and maintaining genomic stability (45). Extracellular HMGB1 interacts with multiple cell-surface receptors, including RAGE, TLR2, TLR4, TLR9, and T-cell immunoglobulin mucin-3 (TIM-3), to activate downstream signaling pathways. These pathways lead to immune cell activation, induction of inflammatory cytokines and type I interferons, promotion of cell adhesion and migration, inhibition of phagocytosis, stimulation of cell proliferation and angiogenesis, and induction of autophagy (79). HMGB1 binds to Toll-like receptors (TLRs), triggering the production of pro-inflammatory cytokines. By inhibiting HMGB1 release and its interaction with TLRs, NAHs reduce the excessive inflammatory response that is often seen in conditions like sepsis, rheumatoid arthritis, and autoimmune diseases (80).

#### 4.1.4 Interaction with chemokines

Host defense relies on the precise coordination of immune cell migration, a process tightly regulated by chemokines. These small signaling proteins orchestrated immune surveillance and inflammatory responses by directing leukocyte movement to specific sites of infection or tissue injury. Beyond their central role in immune cell trafficking, chemokines also govern lymphocyte development, maturation, and homing, thereby shaping the architecture and function of lymphoid and other immune-related organs (81). The activity of chemokines was mediated not only through their cognate chemokine receptors (CKRs) but also through interactions with glycosaminoglycans such as heparin and related non-anticoagulant derivatives. These interactions facilitated the establishment of chemokine gradients that promote cell migration and impart directionality to immune cell movement (44). Notably, heparin had been shown to directly bind to CXCL12, competitively blocking its interaction with the CXCR4 receptor on tumor cells (82). This CXCL12–CXCR4 axis was known to play a critical role in tumor cell migration and metastasis. Conventional LMWHs, including enoxaparin and tinzaparin (which retain significant anti-Xa activity of 70–120 IU/mg), have demonstrated the ability to attenuate pulmonary metastasis in breast cancer models by disrupting this chemokine–receptor interaction. These findings highlight the potential of heparin-based molecules not only as anticoagulants but also as modulators of chemokine-mediated immune and tumor cell trafficking. Importantly, NAHs (with disrupted AT-III binding) can be engineered to retain or even enhance this anti-metastatic property while eliminating bleeding risk (83).

#### 4.1.5 Neutralization of histones and other DAMPs

Another key function of NAHs is their direct binding to histones, thereby mitigating histone-related toxicity. Histone-induced inflammation, endothelial injury, and thrombosis are contributing factors to the development of multiple organ dysfunction syndrome and can even lead to death in sepsis (84). Elevated histone levels are positively associated with sepsis severity. Recent studies have indicated that circulating histones function as damage-associated molecular patterns (DAMPs) and contribute to the pathogenesis of sepsis through multiple mechanisms. These include inducing calcium influx, directly compromising cell integrity and leading to cell death, damaging endothelial cells while increasing vascular permeability, and binding to Toll-like receptors on macrophages and platelets to trigger inflammatory factor release as well as platelet activation and aggregation. In addition, histones promote fibrin polymerization and inhibit plasmin activity, thereby suppressing fibrinolysis (85). Heparin exhibits strong affinity for histones. It reduces histone-induced cytokine release and activity, calcium influx, heparanase release and enzymatic activity, glycocalyx degradation, endothelial injury, as well as the activation of coagulation pathways (38, 50). 4.1.6 Interaction with the protein in virus.

Besides these proteins, heparin could also bind with the protein in virus and affect the function of virus (86). Coronavirus disease 2019 is a newly emerging infectious disease currently spreading across the world. It is caused by a novel coronavirus, severe acute respiratory syndrome coronavirus 2 (SARS-CoV-2). The spike (S) protein of SARS-CoV-2, which plays a key role in the receptor recognition and cell membrane fusion process. Studies have found that SARS-CoV-2’s spike protein has specific regions that bind to HS, which may help the virus infect cells more efficiently. Interestingly, heparin (a similar but more highly sulfated molecule than HS) can block this process by competing with HS for binding to the virus. Experiments show that heparin sticks very tightly to SARS-CoV-2’s spike protein—much more strongly than to other coronaviruses like SARS or MERS. This binding can even change the shape of the spike protein, potentially making it harder for the virus to infect cells. Other studies confirm that heparin-based drugs (like LMWH) can also block the virus, but regular heparin works better because of its longer chain and extra sulfate groups. The inhibitory effect is concentration-dependent, with higher heparin concentrations producing more pronounced blocking activity in cell-based assays (87).

UFH binds to the SARS-CoV-2 spike protein with high affinity (Kd ~40–70 pM), requiring a minimum chain length of an octasaccharide (dp8) for detectable binding (52, 88). NAHs, however, have reduced chain lengths (e.g., roneparstat dp8–16; ODSH dp4–10) and altered sulfation patterns. Based on the chain-length dependency established for UFH, NAHs with average dp >8 may retain partial spike-binding activity, whereas shorter or desulfated derivatives are likely to have substantially reduced affinity. Crucially, direct binding or antiviral activity data for bona fide NAHs are not available. Therefore, while NAHs are hypothesized to exert antiviral effects based on structural similarity, the evidence is currently extrapolated from UFH/LMWH studies and requires NAH-specific validation.

#### 4.1.6 NPP1 and the adenosinergic axis

Ectonucleotide pyrophosphatase/phosphodiesterase-1 (NPP1, CD203a) hydrolyzes ATP to AMP, which is converted to adenosine by CD73. Adenosine signaling through A_2_A receptors promotes immunosuppression, including Treg differentiation and inhibition of effector T cells.

Lopez et al. (89) demonstrated that UFH and LMWHs are potent, selective allosteric inhibitors of NPP1, with UFH showing an IC_50_ of 0.503 µM. Importantly, the study included several NAH chemotypes—including glycol-split, 2-O-desulfated, and 6-O-desulfated heparins—and found that NPP1 inhibition is dissociable from anticoagulant activity, with IC_50_ values ranging from 0.5 µM to 73 µM depending on sulfation pattern. While direct data for NAHs such as roneparstat or ODSH were not reported, the structure-activity relationship established in this study—particularly the finding that charge density (sulfation pattern) rather than chain length is the principal determinant of NPP1 inhibition—suggests that appropriately modified NAHs would retain this activity. This positions NAHs as potential modulators of the adenosinergic axis, a highly active area in cancer immunotherapy.

### 4.2 Inhibition of key pathways

#### 4.2.1 Inflammatory diseases

NAHs exert pleiotropic biological effects by modulating several keys signaling pathways involved in immune regulation and tumor progression. In the context of sepsis, these heparins disrupted the interaction between high mobility group box 1 protein (HMGB1) and lipopolysaccharide (LPS), a pivotal event that drives caspase-11 activation and the subsequent inflammatory cascade.

Recent evidence had established the caspase-11 pathway as a major determinant of septic lethality. In murine models, the caspase-11 pathway has been established as a major determinant of septic lethality. In humans, the functional orthologs are caspase-4 and caspase-5, which perform analogous roles in non-canonical inflammasome activation (39, 90). Caspase-11 was expressed in diverse cell types, including macrophages and endothelial cells. Upon sensing intracellular LPS, caspase-11 cleaves gasdermin D to generate N-terminal fragments that oligomerize and form nanopores in the plasma membrane. This process resulted either in pyroptosis—a lytic and highly inflammatory form of programmed cell death—or in the hyperactivation of surviving cells that release interleukin-1 (IL-1) (91).

During endotoxemia or sepsis, HMGB1 serves as a critical facilitator of LPS-induced caspase-11 activation. LPS stimulation triggers the release of HMGB1 from hepatocytes, which represent the predominant source of circulating HMGB1 during sepsis. In the extracellular milieu, HMGB1 binds to LPS and mediates its uptake into myeloid and endothelial cells through a receptor for advanced glycation end products (RAGE)-dependent endocytic pathway. Subsequent HMGB1-induced lysosomal rupture enables cytosolic translocation of LPS, culminating in caspase-11 activation and amplification of inflammatory signaling (92).

The polysaccharide moiety of LPS played a crucial role in its association with HMGB1. NAHs, which possess high affinity for HMGB1, competitively interfered with this interaction. By preventing the formation of the HMGB1–LPS complex, these heparins effectively blocked cytosolic LPS delivery and suppress caspase-11 activation. Consequently, they attenuate excessive cytokine release, preserve endothelial integrity, and improve vascular function and organ perfusion in severe sepsis. Collectively, these findings highlight the therapeutic potential of NAHs as modulators of the HMGB1–caspase-11 axis in sepsis (93).

#### 4.2.2 Cancer metastasis

NAHs exert multifaceted anti-tumor effects by targeting angiogenic and metastatic signaling pathways. By antagonizing heparin-binding growth factors such as VEGF and bFGF, these agents suppressed angiogenesis and deprive tumors of the nutrients and oxygen required for growth and dissemination (94).

In many carcinomas, particularly those of epithelial origin, lymphatic metastasis occurs more frequently than hematogenous spread during early stages of dissemination (95), highlighting the importance of lymphangiogenic signaling in cancer progression. Among these, the vascular endothelial growth factor C (VEGF-C)/VEGF receptor 3 (VEGFR-3) axis played a central role (96). VEGF-C, secreted by tumor and stromal cells, bound to VEGFR-3 on lymphatic endothelial cells, inducing receptor dimerization and autophosphorylation and activating downstream PI3K/Akt and MAPK/ERK pathways (97). These cascades promoted lymphatic endothelial proliferation, migration, and tube formation, ultimately driving lymph angiogenesis. Overactivation of the VEGF-C/VEGFR-3 pathway facilitated lymphatic metastasis and correlated with poor prognosis in multiple malignancies (95). Conventional LMWHs have also been shown to suppress VEGF-C-induced VEGFR-3 phosphorylation, thereby inhibiting lymphangiogenesis. LMWHs counteracted this process by suppressing VEGF-C–induced VEGFR-3 phosphorylation and thereby inhibiting the formation of new lymphatic vessels. (98, 99). However, unlike NAHs, LMWHs retain clinically significant anticoagulant activity. The observation that LMWHs exhibit anti-metastatic effects independently of anticoagulation supports the rationale for developing true NAHs that isolate these beneficial properties without bleeding risk.

Beyond their anti-(lymph)angiogenic actions, heparin derivatives modulated several pro-metastatic mechanisms. They disrupted platelet–tumor cell interactions that promote hematogenous metastasis and interfere with multiple heparin-binding signaling axes, including TGF-β1, integrins, CXCL12–CXCR4, and VEGF-C/VEGFR-3. In preclinical models of breast, pancreatic, and lung cancer, these effects collectively reduce tumor burden and metastatic dissemination (100).

Mechanistically, LMWHs bind to the heparin-binding domain of CXCL12, shifting the chemokine equilibrium from monomeric to dimeric forms, thereby diminishing its affinity for CXCR4 and attenuating downstream signaling. Derivatives such as LHTD4 further inhibit the TGF-β1 pathway, reducing cancer cell migration and invasion. Consistently, in colon and liver cancer models, enoxaparin has been shown to downregulate CXCR4 expression and suppress CXCL12-mediated proliferation and adhesion (101).

Collectively, these findings indicated that NAHs and their derivatives such as glycol-split or N-desulfated derivatives, can be designed as multifactorial inhibitors of tumor progression by targeting convergent angiogenic, lymphangiogenic, and chemokine-driven pathways, representing a promising therapeutic strategy to limit metastasis and improve clinical outcomes.

#### 4.2.3 Viral infections and immune modulation

Heparin and its derivatives display potent antiviral activity through multiple complementary mechanisms that interfere with viral attachment, entry, and dissemination. Many viruses, including coronaviruses, utilize both ACE2 and cell-surface HS as coreceptors to facilitate host cell entry (102). Heparin, a highly sulfated analogue of HS, competes for viral binding sites and effectively blocks viral attachment to cell membranes. By mimicking HS, heparin can also induce conformational changes in viral surface glycoproteins such as the SARS-CoV-2 spike protein, reducing its affinity for ACE2 and thereby impairing viral internalization (103).

Beyond preventing initial attachment, heparin suppresses viral spread by inhibiting heparanase, the endoglycosidase responsible for HS degradation (104). Since heparanase activity promotes viral egress and cell-to-cell transmission, its inhibition by heparin limits local viral propagation and tissue invasion. Furthermore, heparin’s high degree of sulfation enhances its electrostatic interaction with viral proteins, explaining the superior antiviral efficacy of longer and more heavily sulfated heparin chains compared with shorter fragments (54).

Another noteworthy point is that heparin has beneficial effects on inflammation associated with COVID-19. Severe COVID-19 is frequently characterized by an IL-6–driven “cytokine storm,” with extensive lung injury and disseminated intravascular coagulation (105). Heparin can bind multiple inflammatory cytokines and reduce their levels; it also inhibits a range of adhesion molecules and heparanase, thereby decreasing leukocyte adhesion and transendothelial migration, and promotes nitric oxide release through several pathways. *In vitro*, heparin directly attenuates activation of the nuclear factor-κB (NF-κB) signaling pathway in lipopolysaccharide-stimulated human endothelial cells and monocytes, a pathway implicated in SARS-related pathology (106). Clinically, retrospective analyses indicate that LMWH can significantly reduce plasma IL-6 levels in patients with COVID-19. Based on these anti-inflammatory properties, heparin has also been proposed as a potential therapy for COVID-19–associated myocarditis. Notably, levels of heparin-binding protein (HBP; a 37-kDa cationic antimicrobial protein) rise in critically ill patients as disease worsens; this correlation suggests a role for HBP in systemic inflammation and highlights it as both a disease indicator and a potential therapeutic target in COVID-19 (13).

These actions position heparin as a multifunctional antiviral molecule—one that not only disrupts the early steps of viral attachment and entry but also hinders subsequent dissemination within host tissues. Such properties highlight the potential of heparin and related polysaccharides as promising adjunctive agents in the treatment and prevention of viral infections, including SARS-CoV-2.

### 4.3 Structure-activity relationships of non-anticoagulant heparins

The structure–activity relationship (SAR) of NAHs defines their therapeutic potential by linking precise chemical modifications to distinct biological outcomes. Structural parameters—including sulfation pattern, molecular weight, and backbone flexibility—govern their affinity for various heparin-binding proteins and determine specificity toward targets involved in inflammation, cancer progression, and immune regulation (107). In general, highly sulfated derivatives with higher molecular weight exhibit stronger binding affinities, whereas N-acetylated and glycol-split derivatives show reduced anticoagulant activity but retain nanomolar to micromolar affinity for heparanase and selectins (64, 65, 71) (**Table 1**).

**Table 1:** Structure-activity relationships of heparin derivatives: structural modifications and biological activities.

Modification	Representative compound	MW (kDa)	Anti-Xa (IU/mg)	Heparanase inhibition	P-selectin inhibition (IC_50_)	HMGB1 binding	Key features
None (unmodified)	UFH	15–20	180–200	IC_50_ ~10–50 nM	~40 µg/mL	+++	Potent anticoagulation; high bleeding risk
Depolymerization	Enoxaparin (LMWH)	4.5	90–110	IC_50_ ~1–5 µM	~100 µg/mL	++	Reduced anticoagulation; residual bleeding risk
N-desulfation/N-acetylation	N-acetyl heparin	~15	<20	IC_50_ ~1–5 µM	~175 µg/mL	++	Markedly reduced anticoagulation
Glycol-splitting	RO-heparin	15–18	<20	~95% inhibition at 1 µg/mL	20–60 µg/mL	++	Enhanced flexibility; increased heparanase inhibition
N-acetylation + glycol-splitting	Roneparstat (100NA-ROH)	~16	<10	IC_50_ ~3 nM	>250 µg/mL	++	Highly selective heparanase inhibitor
2-O,3-O-desulfation	ODSH (CX-01)	8–15	<10	IC_50_ ~1–5 µM	>250 µg/mL	+++	Retains HMGB1 neutralization; anti-inflammatory
Partial N-acetylation	58% NA-heparin	~15	~20	~40% inhibition at 1 µg/mL	~25 µg/mL	++	Retains selectin inhibition

UFH, unfractionated heparin; LMWH, low-molecular-weight heparin; ODSH, 2-O,3-O-desulfated heparin; NA, N-acetylated; MW, molecular weight; IC_50_, half-maximal inhibitory concentration; Kd, equilibrium dissociation constant; +++, strong binding; ++, moderate binding. Data sources: 50, 64–66, 71, 108.

IC_50_ values for heparanase inhibition and P-selectin inhibition are derived from different assay formats (enzymatic *vs*. cell adhesion) and are not directly comparable across targets. Anti-Xa activity is expressed in International Units (IU) per milligram, with values <10 IU/mg indicating clinically negligible anticoagulant activity. HMGB1 binding is qualitatively indicated (+++ strong, ++ moderate) due to variations in reported Kd values across different experimental conditions.

#### 4.3.1 Structural modifications and functional selectivity

Targeted desulfation and oxidation can selectively attenuate anticoagulant activity while preserving or enhancing non-anticoagulant functions (109). For example, N-desulfation diminishes interaction with AT-III, effectively abolishing anticoagulation yet maintaining strong affinity for P-selectin and heparanase—key mediators in inflammation and metastasis. Likewise, periodate oxidation removes anticoagulant capacity but retains the ability to modulate immune responses and inhibit tumor cell invasion (49).

#### 4.3.2 Heparanase inhibition and molecular determinants

NAHs act as potent heparanase inhibitors by structurally mimicking HS motifs essential for enzyme recognition while eliminating procoagulant domains. SAR studies reveal that selective 2-*O* and 6-*O*-sulfation, a moderate level of N-acetylation (<50%), and the presence of glycol-split (gs) residues are critical determinants for strong inhibition (110). Glycol splitting at GlcA or IdoA residues conferred greater chain flexibility, optimizing molecular fit within the heparanase catalytic groove. Derivatives such as 2-*O*-desulfated heparin (ODSH) and N-acetylated glycol-split heparins (e.g., Roneparstat/100NA-ROH) exhibit nanomolar IC_50_ values against heparanase, producing robust anti-metastatic and anti-inflammatory effects *in vivo* without anticoagulant or bleeding risks (111).

Higher molecular weight derivatives (Mw > 10 kDa) displayed multivalent interactions capable of simultaneously engaging both heparin-binding domains of heparanase, enhancing inhibitory potency. Additional modifications—such as dicarboxylation (DCoxH) or biotinylation—can further improve bioactivity and pharmacokinetic stability without compromising enzymatic inhibition (11).

#### 4.3.3 Anti-inflammatory and immunomodulatory

Distinct structural motifs also confer strong anti-inflammatory activity independent of coagulation. SAR analyses indicate that selective 2-*O*, 6-*O*, and partial N-sulfation are crucial for interactions with chemokines (e.g., CCL2, CXCL8) and adhesion molecules (e.g., selectins, ICAM-1). Moderate N-desulfation combined with re-N-acetylation minimizes anticoagulant effects while maintaining or even augmenting anti-inflammatory potency. Optimal molecular weight in the 3–6 kDa range favors receptor engagement and tissue penetration, promoting selectivity for inflammatory rather than coagulation pathways. Furthermore, appropriately sulfated and flexible NAHs inhibit proteases such as elastase and block leukocyte adhesion and migration—key processes in inflammation (112). In addition, specific sulfation patterns affect binding to galectin-3, a metastasis-promoting lectin. For example, 2-*O*- or 6-*O*-desulfated and N-acetylated heparin derivatives bind galectin-3 and block galectin-3-mediated tumor–endothelial adhesion and angiogenesis (113). Furtherly, Lopez et al. (89) identified NPP1 (CD203a) as a novel target of heparin derivatives. Systematic evaluation of 15 compounds revealed that charge density (sulfation pattern)—rather than chain length—is the principal determinant of NPP1 inhibitory potency. Importantly, the study included several NAH chemotypes (glycol-split, 2-*O*-desulfated, and 6-*O*-desulfated heparins), and fondaparinux — a non-anticoagulant synthetic pentasaccharide—retained significant activity (IC_50_ 4.99 µg/mL). IC_50_ values ranged from 0.503 µM (UFH) to 73 µM, demonstrating that NPP1 inhibition is dissociable from anticoagulant activity and can be retained in appropriately modified NAHs (89).

#### 4.3.4 Pharmacokinetic and design implications

The molecular size and charge density of NAHs strongly influence their bioavailability and tissue distribution. LMWH and ultra-low-molecular-weight heparin (ULMWH) derivatives exhibit superior absorption and targeted delivery to inflamed or tumor tissues. Collectively, these insights establish a rational SAR framework wherein fine-tuning sulfation pattern, chain length, and backbone flexibility transforms heparin from a traditional anticoagulant into a versatile, non-anticoagulant therapeutic. Such design strategies underpin the development of next-generation heparin-based agents for the treatment of cancer, fibrosis, infection, and inflammatory diseases (114).

## 5 Clinical applications of non-anticoagulant heparins

### 5.1 Sepsis and inflammatory disease

Clinical evidence for NAHs in sepsis remains limited, but retrospective studies and case series have reported encouraging outcomes. For example, a multicenter retrospective study reported that NAHs (**Table 2**) have emerged as promising adjunctive agents in the treatment of sepsis—a life-threatening condition characterized by an uncontrolled inflammatory response leading to endothelial dysfunction, immune dysregulation, and multi-organ failure. Conventional management focuses on infection control and organ support; however, modulation of the host inflammatory response is critical for improving survival outcomes (116).

**Table 2:** Summary of clinical trials.

Compound	NCT Number	Disease	Phase	Primary endpoint	Outcome	Limitations/remarks
Roneparstat	—	Multiple myeloma	I	Safety	Acceptable safety	No dose-limiting toxicity
PG545 + nivolumab	NCT05061017	Advanced solid tumors	II	Objective response rate	Ongoing	Synthetic mimetic, not a NAH
Necuparanib (M402)	—	Metastatic pancreatic cancer	II	Progression-free survival	Ineffective (terminated)	Insufficient efficacy at interim analysis
PI-88	—	Hepatocellular carcinoma	III	Disease-free survival	Not met	Bleeding events
Enoxaparin	—	Small cell lung cancer	III	Overall survival	No improvement (RASTEN)	Conventional LMWH, not a NAH
Dalteparin	—	Lung cancer	III	Overall survival	No improvement (FRAGMATIC)	Conventional LMWH, not a NAH
CX-01	—	Acute myeloid leukemia	II	Response rate	Improved (orphan drug designation)	In combination with chemotherapy

Data sources: 64–66, 71, 115.

Mechanistically, NAHs exert protective effects in sepsis primarily through HMGB1 modulation (see Section 4.1.3 for detailed mechanisms). Clinical studies have shown that adjunctive heparin therapy reduces mortality in septic patients with disseminated intravascular coagulation (92).

Clinical investigations support these mechanistic insights. A multicenter retrospective study reported that adjunctive heparin therapy significantly reduced mortality in patients with disseminated intravascular coagulation (DIC) over a three-month follow-up period. Similarly, controlled clinical trials comparing unfractionated heparin (UFH) or LMWH with placebo demonstrated a reduction in 28-day mortality from 38% to 30% among patients with sepsis or severe sepsis. These benefits were accompanied by improved coagulation profiles, restoration of endothelial proteoglycans, and decreased serum levels of proinflammatory cytokines such as interleukin-6 (IL-6) and tumor necrosis factor-α (TNF-α) (117).

Beyond sepsis, non-anticoagulant and low-molecular-weight heparins exhibit broad organ-protective properties that extend to pulmonary, renal, and pancreatic disorders. In respiratory diseases such as chronic obstructive pulmonary disease (COPD) and asthma, they modulate cytokine release, suppress immune cell infiltration, and reduce mucus hypersecretion, leading to improved pulmonary function and diminished airway hyperresponsiveness. In renal disease, LMWH protects the glomerular filtration barrier by inhibiting heparanase and elastase-mediated matrix degradation, thereby reducing proteinuria and promoting remission in diabetic nephropathy and steroid-sensitive nephrotic syndrome.

In acute pancreatitis, where inflammation, microthrombosis, and endothelial injury converge, LMWH exerts multifactorial benefits. By attenuating inflammation, reducing coagulation abnormalities, and inhibiting pancreatic enzyme activation, LMWH significantly decreases mortality, complications, and hospital stay, while lowering the incidence of pancreatic necrosis. In hypertriglyceridemia-induced pancreatitis, combination therapy with LMWH and insulin further accelerates triglyceride clearance via lipoprotein lipase activation (118).

Finally, these mechanistic and clinical findings underscore the multidimensional therapeutic potential of heparin and its derivatives. Their combined anti-inflammatory, antithrombotic, and organ-protective properties position them as valuable adjuncts in the management of sepsis and related inflammatory disorders, and warrant further translational and clinical investigation (119).

### 5.2 Cancer therapy

NAHs have attracted considerable attention for their anti-metastatic and anti-angiogenic activities, positioning them as promising adjuncts in cancer therapy. By inhibiting heparanase—the key endoglycosidase responsible for degrading HS in the ECM—NAHs prevent ECM breakdown and suppress tumor cell invasion and metastasis (14).

In preclinical cancer models, NAHs significantly reduced tumor burden and metastatic dissemination in breast, pancreatic, and lung cancers. When combined with chemotherapeutic agents, they enhanced therapeutic efficacy while attenuating systemic toxicity, highlighting their potential in combination regimens for both solid and hematologic malignancies (120, 121).

The relationship between cancer and thrombosis was first described by Armand Trousseau in 1865, and venous thromboembolism (VTE) remains the most frequent complication of cancer-associated thrombosis (CAT) (122). LMWHs have long been standard therapy for CAT, but accumulating evidence suggests that heparin and its derivatives also directly modulate tumor biology (123). Through interactions with heparanase, P-/L-selectins, and the chemokine (C-X-C motif) ligand 12 (CXCL12) and its receptor CXCR4 (CXCL12–CXCR4) chemokine axis, heparins interfere with key processes governing tumor cell signaling, inflammation, angiogenesis, and metastasis (71). A direct side-by-side comparison of selectin-specific versus heparanase-specific NAH derivatives in MC38 colon carcinoma and B16-BL6 melanoma metastasis models is provided by Bendas & Borsig (77), who showed that distinct non-anticoagulant heparin modifications can preferentially target different adhesion axes (selectins *vs*. integrins *vs*. heparanase), highlighting the importance of target-specific NAH design.

However, because LMWHs retain significant anti-Xa activity, their use in cancer patients carries a bleeding risk. Chemically modified NAHs with minimal anticoagulant activity retain potent anticancer properties. For example, 2- or 6-*O*-desulfated and N-acetylated derivatives bind galectin-3 (a metastasis-promoting lectin) and then block galectin-3-mediated tumor–endothelial adhesion and angiogenesis without cytotoxicity (113). Such derivatives represent promising templates for next-generation anti-metastatic drugs that lack the bleeding risk associated with LMWHs. Similarly, several heparin mimetics, including PI-88 and its second-generation analogue pixatimod (PG545), inhibit heparanase and multiple angiogenic factors such as VEGF and FGF. Notably, PG545 also exerts immunostimulatory effects through a distinct, heparanase-independent mechanism: it activates plasmacytoid dendritic cells via Toll-like receptor 9 (TLR9), inducing type I interferon (particularly IFN-α) production and natural killer cell activation (115). PG545 has demonstrated favorable pharmacokinetics, immunomodulatory activity, and disease stabilization in early clinical studies, and is currently under Phase II evaluation in combination with nivolumab (NCT05061017) (124). Despite encouraging preclinical data, clinical outcomes of heparin derivatives in oncology have been mixed. The RASTEN Phase III trial (125) investigated enoxaparin as adjunctive therapy in small-cell lung cancer and found no significant improvement in overall survival. Similarly, the FRAGMATIC trial (126) failed to demonstrate survival benefit with dalteparin in over 2,000 lung cancer patients, while noting an increased risk of clinically relevant bleeding. These negative results underscore the importance of distinguishing the anticoagulant effects of conventional LMWHs from the potential non-anticoagulant activities that NAHs aim to exploit.

Among true NAHs, the heparin mimetic necuparanib (M402) advanced to Phase II trials for metastatic pancreatic cancer but was discontinued after interim futility analysis due to insufficient efficacy (127). Likewise, PI-88, a heparanase-inhibiting heparin mimetic, failed to meet its primary disease-free survival endpoint in a Phase III trial for hepatocellular carcinoma and was associated with bleeding complications (124). These failures highlight several challenges: the difficulty of translating potent *in vitro* heparanase inhibition into clinical antitumor efficacy; the likely requirement for optimal patient selection (e.g., biomarker-driven); and the persistent bleeding risk, albeit reduced compared to UFH, which remains a concern for certain heparin derivatives.

In contrast, some NAHs have shown more encouraging results in non-oncology indications. Roneparstat (SST0001) demonstrated acceptable safety in a Phase I trial for multiple myeloma and is proceeding to Phase II evaluation (128). The 2-O,3-O-desulfated heparin derivative CX-01 has received Orphan Drug designation for acute myeloid leukemia based on improved remission rates when combined with chemotherapy (129). Collectively, these contrasting outcomes suggest that the therapeutic potential of NAHs may be indication-dependent, and that rigorous, hypothesis-driven clinical trial design—incorporating appropriate biomarkers and patient selection—is essential for future success. (125).

Heparin-induced thrombocytopenia (HIT) is an immune complication driven by antibodies against platelet factor 4 (PF4)/heparin complexes, and the risk correlates with heparin’s sulfation density and chain length (130). Since NAHs are engineered with altered sulfation and reduced molecular weight, their HIT risk is expected to be lower than that of UFH. For example, the 2-*O*,3-*O*-desulfated derivative ODSH (CX-01) has not been associated with HIT in clinical studies to date (129). Notably, fondaparinux — a synthetic pentasaccharide — has negligible HIT risk, suggesting that sufficiently short or desulfated NAHs may also be safe in this regard. However, direct evidence for most NAH derivatives remain limited, and prospective safety evaluation is needed. Looking forward, the identification of NPP1 as a target of heparin derivatives (89) opens a potential new direction for NAH-based cancer immunotherapy. Notably, NPP1 inhibition was dissociable from anticoagulant activity, and several NAH chemotypes (glycol-split, 2-O-desulfated, 6-O-desulfated heparins) were active. Although direct data for roneparstat, ODSH, or other true NAHs are not yet available, the established SAR—particularly the importance of sulfation pattern over chain length—provides a rational basis for designing NAHs optimized for NPP1 inhibition. This mechanism could synergize with immune checkpoint inhibitors and warrants systematic investigation in future studies.

Overall, extensive experimental and early-phase clinical evidence supports the multifaceted antitumor potential of heparin and its derivatives. Continued efforts are needed to optimize structural modifications that preserve anticancer and anti-metastatic functions while minimizing anticoagulant effects. Several factors may explain the negative outcomes. First, patient selection — the RASTEN and FRAGMATIC trials enrolled unselected lung cancer patients without biomarker stratification. Given that heparanase expression varies widely across tumors and correlates with metastasis, patients with low heparanase expression may not benefit from heparanase-targeting agents. Second, dosing — both trials used anticoagulant doses (enoxaparin 1 mg/kg, dalteparin 5000 IU), which may be insufficient for non-anticoagulant effects. Dose escalation is limited by bleeding risk, highlighting the advantage of true NAHs. Third, mechanism — the anti-metastatic effects of heparins may be most relevant in the perioperative period, whereas these trials administered LMWH for weeks to months, potentially missing the critical window of intervention. Future research should emphasize mechanistic elucidation, rational dosing strategies, and combinatorial regimens with immunotherapy and chemotherapy to fully realize the clinical promise of NAHs in oncology.

### 5.3 Anti-viral applications

The antiviral potential of NAHs has gained increasing recognition as a novel strategy for combating viral infections. These derivatives inhibit viral entry by binding to viral surface glycoproteins and preventing their interaction with host cell receptors, thereby blocking the initial step of infection and limiting viral replication.

Beyond entry inhibition, NAHs can modulate host immune responses, enhancing viral clearance through immunoregulatory effects. This dual mechanism—direct viral blockade and immune modulation—renders NAHs promising candidates against a range of viruses, including influenza, HIV, and SARS-CoV-2.

In particular, studies have shown that NAHs effectively prevent SARS-CoV-2 infection by binding to the viral spike protein and disrupting its interaction with the angiotensin-converting enzyme 2 (ACE2) receptor on human cells. By interfering with this essential entry step, NAHs may serve as adjunctive agents in the early treatment of COVID-19, reducing viral spread and disease progression. Notably, while conventional LMWHs also exhibit some antiviral activity, they simultaneously possess anticoagulant effects that are absent in true NAHs. By interfering with this essential entry step without anti-Xa activity, NAHs may serve as safer adjunctive agents in the early treatment of COVID-19, reducing viral spread and disease progression without bleeding risk.

These findings highlight the broad-spectrum antiviral properties of NAHs and their potential role in future antiviral therapeutics (16).

### 5.4 Vascular health and cardiovascular disease

NAHs exert significant vascular-protective effects, particularly in conditions characterized by endothelial dysfunction. These compounds enhance endothelial eNOS activity, increasing NO production—an essential mediator of vasodilation, vascular homeostasis, and thrombosis prevention. Through improved NO bioavailability, NAHs help maintain vascular tone, inhibit platelet aggregation, and reduce the risk of vascular occlusion (59).

Notably, the angiogenic effects of NAHs are context-dependent, exerting opposing outcomes in tumor versus post-ischemic settings. To clarify this duality, we summarize the distinct features of NAH-mediated angiogenesis in these two contexts (**Table 3**). It is important to distinguish the context-dependent effects of NAHs on angiogenesis. In the tumor microenvironment, angiogenesis is driven by pathologically high concentrations of pro-angiogenic factors such as VEGF and bFGF, which are often overexpressed by hypoxic cancer cells. NAHs inhibit this process by competing with heparan sulfate proteoglycans for growth factor binding, thereby disrupting growth factor-receptor interactions and downstream signaling. This anti-angiogenic effect is desirable for limiting tumor neovascularization and metastasis. In contrast, post-ischemic cardiac repair involves physiological angiogenesis in response to tissue hypoxia, where VEGF and bFGF levels are tightly regulated and temporally controlled. In this setting, NAHs exert protective effects primarily through eNOS activation and NO production which preserve endothelial function, promote vasodilation, and support the formation of stable, functional collateral vessels. Additionally, NAHs may stabilize endogenous growth factors, prolonging their bioavailability at the site of ischemia without overstimulating pathological angiogenesis.

**Table 3:** Context-dependent effects of NAHs on angiogenesis.

Feature	Tumor angiogenesis	Post-ischemic cardiac angiogenesis
Pathological/physiological	Pathological	Physiological (repair)
Growth factor levels	Constitutively elevated	Transiently increased
Primary mechanism	Competitive inhibition of VEGF/FGF signaling	eNOS activation and NO production
Net effect of NAHs	Anti-angiogenic	Pro-angiogenic
Therapeutic goal	Suppress neovascularization	Support revascularization

In cardiovascular disease, NAHs have shown potential benefits in post-myocardial infarction therapy. By promoting endothelial repair and stimulating angiogenesis, they support vascular regeneration and improve myocardial perfusion. These mechanisms may mitigate long-term complications such as heart failure and recurrent ischemic events (131).

The ability of NAHs to restore endothelial function and enhance vascular integrity underscores their promise as adjunctive agents in cardiovascular disease management. Nevertheless, direct evidence for true NAHs (periodate-oxidized or N-desulfated derivatives) in cardiovascular settings remains limited. Most human data derive from UFH or LMWH rather than bona fide NAHs. Thus, while the eNOS/NO pathway is well-established for heparin derivatives in general, NAH-specific cardiovascular validation remains an open area for future investigation.

## 6 Pharmaceutical application of non-anticoagulant heparins

**Figure 6:**
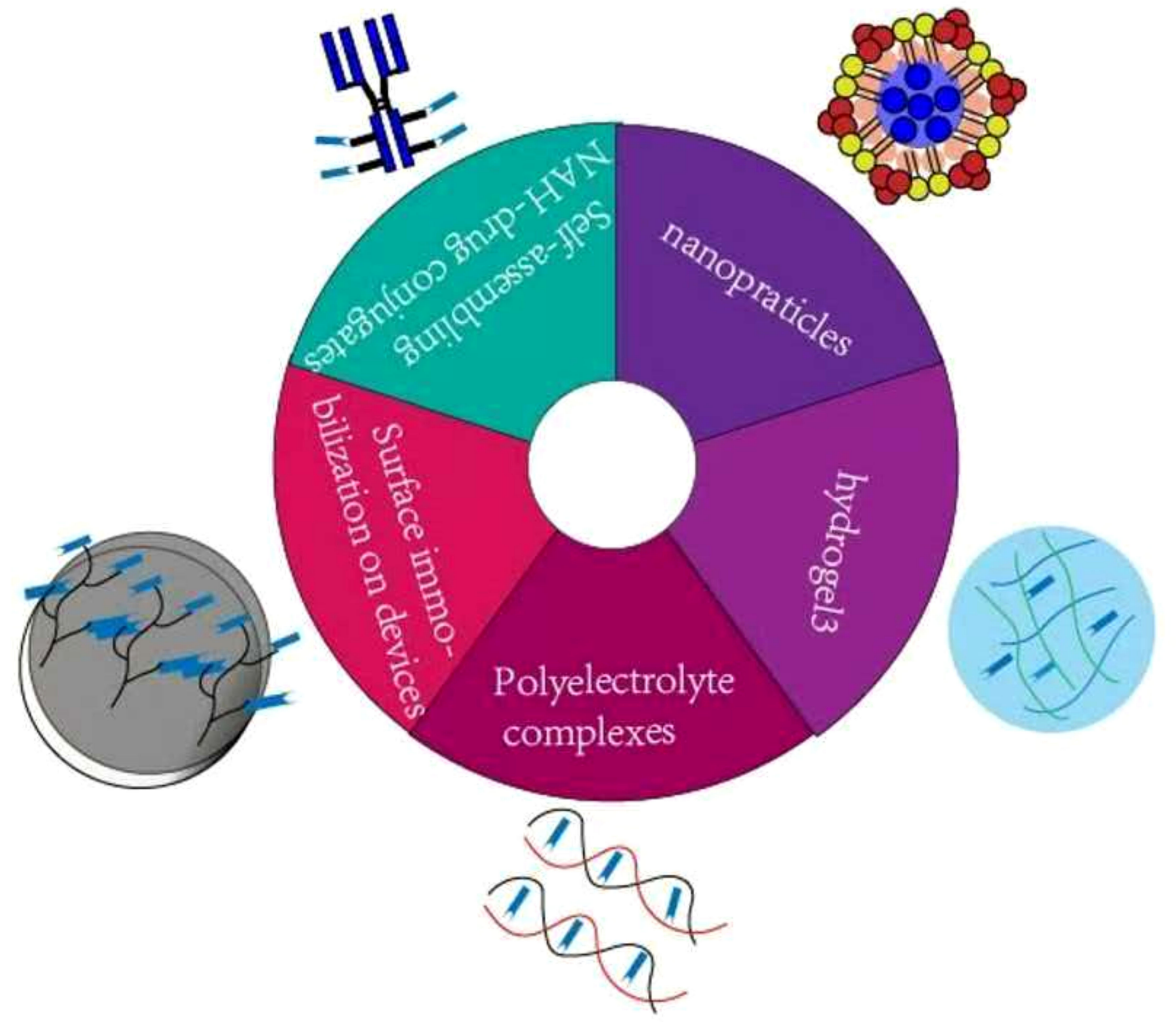
Schematic illustration of heparin derivatives and their applications. **(A)** Self-assembling NAH-drug conjugates; **(B)** NAH-containing hydrogels for localized release; **(C)** Polyelectrolyte complexes with protamine; **(D)** Surface immobilization on medical devices; **(E)** Ligand-decorated targeted nanoparticles. This image is created by the authors using Powerpoint.

NAHs are increasingly leveraged as pharmaceutical building blocks across drug delivery, oncology, and tissue engineering owing to their biocompatibility, biodegradability, and high affinity for heparin-binding proteins. Their high charge density, hydrophilicity, and relatively large hydrodynamic size, however, can drive rapid clearance, limited tissue penetration, and colloidal instability. Contemporary formulation strategies therefore combine structure tailoring (e.g., selective desulfation, glycol-splitting, prodrug conjugation) with delivery technologies (nanoparticles, hydrogels, polyelectrolyte complexes, surface immobilization) to optimize pharmacokinetics, pharmacodynamics, and safety while minimizing residual anticoagulant activity (**Figure 6**) (132–135). However, the translational feasibility of these formulation strategies varies considerably. This section summarizes current formulation approaches and, where available, discusses pharmacokinetic properties, stability data, manufacturability, regulatory status, and clinical development stage to provide a practical perspective on clinical translation.

### 6.1 Self-assembling NAH–drug conjugates

Covalent linkage of hydrophobic payloads (e.g., paclitaxel, doxorubicin) to NAHs via cleavable spacers (valine/leucine, hydrazone, disulfide) yields amphiphiles that self-assemble into ~140–180 nm nanoparticles. These systems enable high loading and circulation stability with sustained or stimulus-responsive release, while the NAH segment confers anti-angiogenic and anti-heparanase co-benefits. Spacer chemistry, substitution density, and NAH chain length was tuned to balance carrier stability with on-target release and intratumoral accumulation. Heparin–cisplatin constructs enhance cellular uptake and can sensitize tumors to platinum therapy (136, 137).

### 6.2 Hydrogels for localized, prolonged delivery

NAH-containing hydrogels (e.g., heparin–poloxamer matrices) sequester and protect growth factors such as BMP-2, FGF-2, NGF, and HGF through specific affinity interactions, creating depot formulations for long-acting local release. Injectable thermo- or shear-responsive formats support minimally invasive placement, whereas ionic competition or enzymatic remodeling provides controlled liberation of bioactives for bone/cartilage repair and nerve regeneration (138). Heparin–paclitaxel and heparin–doxorubicin conjugates increase intratumoral drug exposure and attenuate systemic toxicity, while the NAH domain interferes with VEGF/FGF signaling and heparanase activity to restrain angiogenesis and metastasis. Chemically modified, non-anticoagulant derivatives that bind galectin-3 inhibit tumor–endothelium adhesion and neovascularization without intrinsic cytotoxicity, supporting anti-metastatic applications (139).

### 6.3 Polyelectrolyte complexes with protamine

Combining LMWH (~5 kDa) with protamine at ~6:4 produces LMWH/protamine nano-/microparticles (~50–200 nm; ζ ≈ –25 to –30 mV). These PECs bind heparin-binding cytokines (e.g., FGF-2, HGF, platelet-rich-plasma factors), stabilize them, and enable controlled release. As coatings and injectable gels, they enhance adhesion and viability of endothelial cells, fibroblasts, adipose-derived stromal/stem cells (ADSCs) and bone marrow-derived mesenchymal stem cells (BMSCs) and CD34^+^ progenitors and have induced vascularization and fibrous tissue formation *in vivo* (140).

### 6.4 Surface immobilization on devices

Covalent grafting of heparin onto stents and scaffolds using EDC/NHS coupling (carboxyl-to-amine) or periodate oxidation followed by reductive amination imparts durable thrombo-resistance and establishes affinity interfaces for controllable factor capture/release. Although early heparin-only coatings did not eliminate restenosis, integration with drug-eluting layers (e.g., paclitaxel) or growth-factor-binding domains shows potential to accelerate re-endothelialization and improve long-term patency (141).

### 6.5 Targeted delivery and precision engineering

Ligand decoration of NAH nanoparticles with antibodies or fragments (e.g., anti-HER2), peptides (RGD, NGR), or aptamers improves tissue-selective uptake and reduces off-target exposure. Prodrug spacers regulate self-assembly and triggerable cleavage, enabling circulation stability with lesion-activated release. Microenvironment-responsive mechanisms (acidic pH, elevated glutathione, protease/heparanase activity) further concentrate payloads at disease sites while limiting systemic toxicity (142, 143).

To provide a practical reference for researchers and clinicians considering NAH-based formulations, we summarize the translational status of the delivery strategies discussed above, including available pharmacokinetic data, scalability, regulatory considerations, and current clinical stage (**Table 4**).

**Table 4:** Translational status of the NAH-based formulation strategies.

Formulation type	PK data available?	Scalability	Regulatory status	Clinical stage	Major barriers
Self-assembling conjugates	Preclinical only	Lab scale	None	Preclinical	Scale-up, batch consistency
Hydrogels	Preclinical only	Lab to pilot	None	Preclinical (except non-NAH analogs)	Sterilization, degradation kinetics
Polyelectrolyte complexes	Preclinical only	Lab scale	None (protamine FDA-approved)	Preclinical	Batch variation, protamine sourcing
Surface immobilization	Extensive (leaching studies)	Industrial scale	FDA-approved for heparin (not NAH)	Clinical (heparin-coated devices)	NAH-specific approval needed
Targeted nanoparticles	Preclinical only	Lab scale	None	Preclinical	Manufacturing complexity, ligand stability

### 6.6 Remaining challenges and unresolved questions

Despite promising preclinical data, several barriers must be overcome before NAHs can be widely adopted.

Structural heterogeneity. NAHs derived from animal-source heparin are inherently heterogeneous mixtures, differing in molecular weight, sulfation pattern, and sequence. This poses challenges for batch-to-batch reproducibility and quality control, as highlighted by the 2008 heparin contamination crisis (144). Fully synthetic, sequence-defined heparin oligosaccharides (e.g., fondaparinux) offer precise structural control but are costly and limited to short chains (145). Chemoenzymatic synthesis may bridge this gap (146), but scalability remains a challenge.

Regulatory ambiguity. Neither the FDA nor the EMA has established a clear threshold—such as a specific anti-Xa activity level—that defines a heparin derivative as “non-anticoagulant.” This regulatory gap creates uncertainty for drug developers regarding safety requirements and approval pathways, as NAHs lack the standardized pharmacopoeial monographs that exist for LMWHs.

Route of administration. Most NAHs require parenteral delivery. For acute indications (e.g., sepsis), this is acceptable. However, for chronic inflammatory diseases requiring long-term outpatient use, parenteral delivery is impractical. Inhaled delivery is a promising alternative for pulmonary indications (147), but oral delivery—the most convenient route—remains largely unsolved despite decades of research (148).

## 7 Conclusion and future perspectives

NAHs have emerged as versatile therapeutic scaffolds that modulate disease-critical pathways through glycan-mediated interactions with heparanase, selectins, HMGB1, chemokines, and in some cases TLR9. These mechanisms position NAHs as potential modulators of both innate and adaptive immunity, particularly through effects on macrophage polarization and the tumor immune microenvironment.

However, translational progress has been mixed. Preclinical studies consistently demonstrate anti-tumor, anti-inflammatory, and anti-viral activities, yet clinical outcomes have been disappointing in several late-stage trials (RASTEN, FRAGMATIC, necuparanib, PI-88). These failures highlight deeper challenges: the difficulty of translating *in vitro* potency into clinical efficacy, the need for biomarker-driven patient selection, and the persistent bleeding risk that complicates dose escalation. Additionally, the field lacks a regulatory definition of “non-anticoagulant,” and most NAHs require parenteral delivery—an impractical route for chronic outpatient use.

Looking forward, priorities include: chemistry-driven optimization of target selectivity, biomarker development for patient stratification, systematic evaluation of combination strategies (e.g., with checkpoint inhibitors), and delivery innovations (inhaled or oral formulations). Realizing the therapeutic potential of NAHs will require not only better chemistry but also smarter trial design and a realistic appraisal of both opportunities and limitations.
